# A Roadmap for Optimizing Asthma Care Management via Computational Approaches

**DOI:** 10.2196/medinform.8076

**Published:** 2017-09-26

**Authors:** Gang Luo, Katherine Sward

**Affiliations:** ^1^ Department of Biomedical Informatics and Medical Education University of Washington Seattle, WA United States; ^2^ College of Nursing University of Utah Salt Lake City, UT United States

**Keywords:** patient care management, clinical decision support, machine learning

## Abstract

Asthma affects 9% of Americans and incurs US $56 billion in cost, 439,000 hospitalizations, and 1.8 million emergency room visits annually. A small fraction of asthma patients with high vulnerabilities, severe disease, or great barriers to care consume most health care costs and resources. An effective approach is urgently needed to identify high-risk patients and intervene to improve outcomes and to reduce costs and resource use. Care management is widely used to implement tailored care plans for this purpose, but it is expensive and has limited service capacity. To maximize benefit, we should enroll only patients anticipated to have the highest costs or worst prognosis. Effective care management requires correctly identifying high-risk patients, but current patient identification approaches have major limitations. This paper pinpoints these limitations and outlines multiple machine learning techniques to address them, providing a roadmap for future research.

## Introduction

Asthma affects 9% of Americans [1-3] and incurs US $56 billion in cost [4], 3630 deaths, 439,000 hospitalizations, and 1.8 million emergency room visits annually [1]. As is true for many chronic diseases, a small fraction of asthma patients with severe disease, high vulnerabilities, or great barriers to care consume most health care costs and resources [5,6]. The top 20% of patients consume 80% of costs, and the top 1% consume 25% [6,7]. An effective approach is needed to find high-risk patients and implement appropriate interventions to improve outcomes and to reduce costs and resource use.

Almost all private health plans provide, and most major employers purchase, care management services that implement tailored care plans with early interventions for high-risk patients to avoid high costs and health status degradation [8-10]. Care management is a cooperative process to assess, plan, coordinate, implement, evaluate, and monitor services and options to fulfill a patient’s health and service needs [11]. It includes a care manager who regularly calls the patient, arranges for health and related services, and helps make medical appointments. Asthma exacerbations account for 63% of annual total asthma cost [12,13]. Using care management properly can reduce asthma exacerbations, cut hospital admissions and emergency room visits by up to 40% [9,14-18], trim cost by up to 15% [15-19], and enhance patient treatment adherence, quality of life, and satisfaction by 30% to 60% [14].

Although widely used, care management has costs of its own and can require more than US $5000 per patient per year [15]. Owing to resource constraints, usually only 1% to 3% of asthma patients are enrolled in care management [7]. Ideally, the ones enrolled should be those at the highest risk. Predictive modeling is the best method to find high-risk patients [20]. It uses a model for predicting individual patient cost or health outcome to automatically find high-risk patients [14,21-26]. Cost reflects use and efficiency of care and indirectly reflects outcomes such as hospitalization and emergency room visit. For patients predicted to have the highest costs or worst outcomes, care managers examine patient records, consider various factors such as social ones, and make the ultimate allocation and intervention decisions. Correct identification of high-risk patients is key to effective care management, but current identification methods have limitations. This paper makes two contributions. First, we pinpoint these limitations. Second, we outline several machine learning techniques to address them, offering a roadmap for future research. Clinical machine learning is a promising technology for finding high-risk patients [27]. Our discussion focuses on the machine learning predictive modeling aspect of care management for identifying high-risk patients. Besides this, several other factors such as patient behavior pattern, patient motivation, trigger for patient engagement [28-30], and patient and caregiver education [31] also impact a care management program’s performance. A detailed discussion of how to incorporate or change these factors to optimize asthma care management is beyond this paper’s scope.

## Limitations of Current Patient Identification Methods for Asthma Care Management

### Limitation 1: Low Prediction Accuracy Causes Misclassification, Unnecessary Costs, and Suboptimal Care

Current predictive models for individual patient costs and health outcomes exhibit poor accuracy causing misclassification and need improvement. When projecting individual patient cost, the *R*^2^ accuracy measure of models reported in the literature is less than 20% [32], and the average error is typically comparable to the average cost [33]. When projecting individual patient health outcome, the area under the receiver operating characteristic curve accuracy measure is usually much smaller than 0.8 [6,34-40]. Those large errors make enrollment miss more than half of patients a care management program can help most [14,26]. Weir et al [26] showed that the top 10% risk group identified by a predictive model missed more than 60% of the top 10% and about 50% of the top 1% of patients who had the largest costs. If we could find 10% more of the top 1% patients who had the largest costs and enroll them, we could save up to US $210 million in asthma care each year and also improve health outcomes [6,36,37]. In general, because of the large patient base, a small improvement in accuracy will benefit many patients, having a large positive impact. A 5% absolute improvement in accuracy already makes a health care system willing to deploy a new model [41].

Existing predictive models have low accuracy for multiple reasons, which include the following:

1. Although several dozen risk factors for adverse outcomes in asthma are known [6,18,36,39,40,42-46], an existing model typically uses only a few of them (eg, less than 10) [6,36-39]. Existing models were often constructed using data obtained from clinical trials or old-fashioned electronic medical records (EMRs) that collected only a limited set of variables [47]. No published model explores all known risk factors available in modern EMRs, which collect an extensive set of variables [47].
2. As with many diseases, many features (also known as independent variables that include both raw and transformed variables) predictive of adverse outcomes in asthma have likely not been identified. For instance, using a data-driven approach to find new predictive features from many variables in EMRs, Sun et al [48] improved prediction accuracy of heart failure onset by more than 20%. Existing predictive models for health outcomes of individual asthma patients were developed mainly using a small number of patients (eg, <1000) or variables (eg, <10) [6,36-39], creating difficulty in finding many predictive features and their interactions.
3. Existing models mainly use patient features only, presuming that each patient’s cost and health outcomes relate only to the patient’s characteristics and are unassociated with characteristics of the health care system (eg, the treating physician and facility). However, system features are known to be influential, have larger impacts on patients with the worst outcomes, and can account for up to half of the variance in their outcomes in certain cases [49-52]. The use of physician characteristics has been examined in predictive modeling only minimally [35], creating a knowledge gap for system features in general.
4. Applying care management to a patient tends to improve the patient’s health outcomes and reduce the patient’s cost, excluding the cost of care management. Yet, existing models omit the factor of care management enrollment.
5. A health care system often has limited training data, whereas a model’s accuracy generally increases with more training data. Different systems have differing data distributions [53] and collected attributes, impacting the performance and applicability of a model trained using one system’s data for another system [54-57]. To address these two issues, one can perform transfer learning and use other source systems’ information to improve model accuracy for the target system [54,58,59]. Transfer learning typically requires using other source systems’ raw data [60,61]. However, systems are rarely willing to share their raw data because of confidentiality concerns with regard to patient data. Research networks [62-64] mitigate, but do not solve, the problem. Many systems are outside a network, whereas systems in it share raw data of limited attributes. Alternatively, one can conduct model updating, model averaging, or ensemble averaging that requires only the trained models, but not the raw data, from other source systems. Model updating applies to only one source system and cannot combine information from multiple source systems, limiting the improvement in model accuracy. Many model updating methods work for only certain kinds of models [65]. Model averaging usually employs the same averaging approach such as weights in all regions of the feature space [66]. Yet, to boost model accuracy, different averaging approaches are often needed in differing regions [67]. Also, if the target system does not have enough data to train a reasonably accurate model as a starting point, further averaging with the trained models from other source systems may not improve the final model’s accuracy to a satisfactory level.

### Limitation 2: No Explanation of the Reasons for a Prediction Causes Poor Adoption of the Prediction and Busy Care Managers to Spend Extra Time and Miss Suitable Interventions

Unlike physicians who see patients regularly, care managers often have no prior interaction with a patient and are unfamiliar with the patient’s medical history when they need to make enrollment decisions. They need to understand why a patient is forecasted to be at high risk before allocating to care management and creating a tailored care plan, but have limited time to review extensive patient records with many variables, possibly accumulated over a long time and often including hundreds of pages [68]. Patients are at high risk for various and often multiple reasons, each linking to one feature or a combination of several features. Each combination represents a risk pattern rather than a risk factor (a single variable) and cannot be found by regular risk factor finding methods. An example risk pattern is that the patient had two or more urgent care visits for asthma last year AND lives 15 miles or more away from the patient’s physician. Complicated predictive models, covering the majority of machine learning models such as random forest, provide no justification for predictions of high risk. This causes poor adoption of the prediction and forces care managers to spend great effort finding root causes, which often involves manual temporal aggregation of clinical variables such as counting urgent care visits. This is time consuming, likely to miss more patients who would gain most from care management, and difficult to do when appropriate cut-off thresholds for numerical variables (eg, 15 miles in distance) are unknown.

Existing predictive models provide limited help in creating tailored care plans. An intervention targeting the reason underlying the high risk is likely to have better effect than nonspecific ones. A patient can have high risk for several reasons. A care manager may develop a tailored care plan for a patient using variable and subjective clinical judgment, but may miss certain suitable interventions because of the following reasons. First, many features exist. As true for any human, a typical care manager can process no more than 9 information items at once [69], making it hard to find all reasons from many possible feature combinations. Second, considerable practice variation such as by 1.6 to 5.6 times appears across differing care managers, facilities, and regions [5,34,70-78]. Third, care managers usually include in the care plan interventions addressing patient factors only. For the health care system, some useful interventions such as extending physician office hours are not identified as possible interventions. Interventions at the system level can be more efficient and effective than those for patients [50,79]. Some system levels, such as treating physicians, are more accessible than patients. An intervention at the system level can affect many patients, whereas an intervention for a patient affects only that patient. Missing suitable interventions degrades outcomes.

### Limitation 3: For Patients on Care Management, a Lack of Causal Inference Capability Causes the Predictive Model to Give No Clear Guidance on Which Patients Could Be Moved off Care Management

An asthma patient’s risk changes over time, whereas a care management program can enroll only limited patients. To best use the program, all patients remaining in the health plan are reevaluated for their risk periodically, for example, on an annual basis. The patients who are in the program and now predicted to be at low risk are moved off the program to make room for those previously at low risk but now at high risk. Doing this properly requires answering intervention queries via causal inference [80,81], which is beyond most existing predictive models’ capability. Some patients in the program are in a stable status and ready to safely leave the program. For some others, using the program is essential for keeping them at low risk. Moving them off the program can lead to undesirable outcomes. An existing model can predict a patient in the program to be at low risk, but often does not tell which of the two cases the patient falls into and does not give clear guidance on whether the patient should be moved off the program. This is particularly the case if we expect care management to have greatly varying impact across different subgroups of patients and would like to consider their differences in impact explicitly.

## Machine Learning Techniques for Optimizing Asthma Care Management

New techniques are needed to identify more high-risk asthma patients and provide appropriate care. Besides those proposed in our paper [27], we can use the following machine learning techniques to optimize asthma care management.

### Techniques for Improving Prediction Accuracy of Individual Patient Costs and Health Outcomes

#### Use All Known Risk Factors for Adverse Asthma Outcomes Available in Modern Electronic Medical Records

Many risk factors for adverse asthma outcomes are known [6,13,36,39,40,42-46] and available in modern EMRs. To fully use their predictive power, we consider all of these risk factors when building models for predicting individual patient costs and health outcomes. We perform feature selection to remove known risk factors that are not predictive for reasons such as data quality and variable redundancy. Clinical experts can suggest for consideration additional features that have clear medical meaning but have not previously been used for predicting asthma outcomes or costs. Two examples of such features are as follows: (1) exercise vital signs and (2) whether the patient has seen an asthma specialist (allergist or pulmonologist) recently. Patients who have seen asthma specialists tend to have more severe asthma, worse health outcomes, and higher costs than those who have seen primary care physicians only. Another way to consider this factor is to build separate models for patients who have seen asthma specialists and patients who have seen primary care physicians only.

#### Use Many Asthma Patients and Patient Features

Many features predictive of adverse outcomes in asthma have not yet been identified. To find new predictive features, we use many asthma patients and a data-driven approach to explore many standard patient features listed in the clinical predictive modeling literature [5,34,65]. Some patient features cover social, economic, and community factors. An example of such features is the average income level of the area that the patient lives in. To combine known risk factors and predictive features derived from data, during feature selection we give a higher weight to known risk factors (eg, by multiplying their scores by a factor larger than 1) so that they are more likely to be selected than the other features. This new approach can handle both categorical and numerical variables, discover new predictive features, and remove known risk factors that are not predictive for reasons such as data quality and variable redundancy. In contrast, the existing method for combining known risk factors and predictive features derived from data [48,82] can neither directly handle categorical variables nor remove known nonpredictive risk factors.

#### Use Health Care System Features

To consider their impact, we include health care system features in building models for predicting individual patient costs and health outcomes. For each health care system level, such as physician or facility, we construct a profile containing its own information (eg, facility hours) and aggregated historical data of its patients (omitting the index patient) extracted from the provider’s administrative and EMR systems. The count of the physician’s asthma patients [83] is an example of profile variables.

Some system features are computed using only system profile variables. Our paper [27] listed several physician-level features such as the average outcome of a physician’s asthma patients. Examples of facility-level features are as follows: (1) whether a facility is open at night or on weekends, (2) the number of staffed beds in a hospital, (3) facility type, and (4) availability of enhanced services such as asthma hotline, foreign language translation, special primary care team for asthma, and special home care. The other system features are computed by combining system profile and patient variables, reflecting the match of physician or facility and patient. An example of such features is the distance between the patient’s home and closest urgent care facility.

#### Use All Patients

The standard approach for predicting individual patient costs or health outcomes in asthma is to build a model using only asthma patient data. In the presence of many features, we may not have enough asthma patients to train the model and to obtain high prediction accuracy. To address this issue, we add a binary indicator feature for asthma and train the model on all patients, not just asthma patients. Asthma patients and other patients share many features in common. We can better tune these features’ coefficients in the model by using all patients.

#### Consider the Factor of Care Management Enrollment

To consider care management’s impact on costs and health outcomes, we add a binary indicator feature for care management enrollment when building models for predicting individual patient costs and health outcomes [84].

#### Perform Transfer Learning Using Trained Models From Other Source Health Care Systems

To address limited training data and improve model accuracy for the target health care system, we perform transfer learning using trained models from other source systems. Organizations are usually more willing to share their trained models than their raw data. Publications often describe trained models in detail. A model trained using a source system’s data includes much information useful for the prediction task on the target system, particularly if the source system has lots of training data. Our transfer learning approach can handle all kinds of features, prediction targets, and models used in the source and target systems. Our approach can potentially improve model accuracy regardless of the amount of training data available at the target system. Even if the target system has enough training data in general, it may not have enough training data for a particular pattern. A trained model from a source system can help make this up if the source system contains enough training data for the pattern.

**Figure 1:**
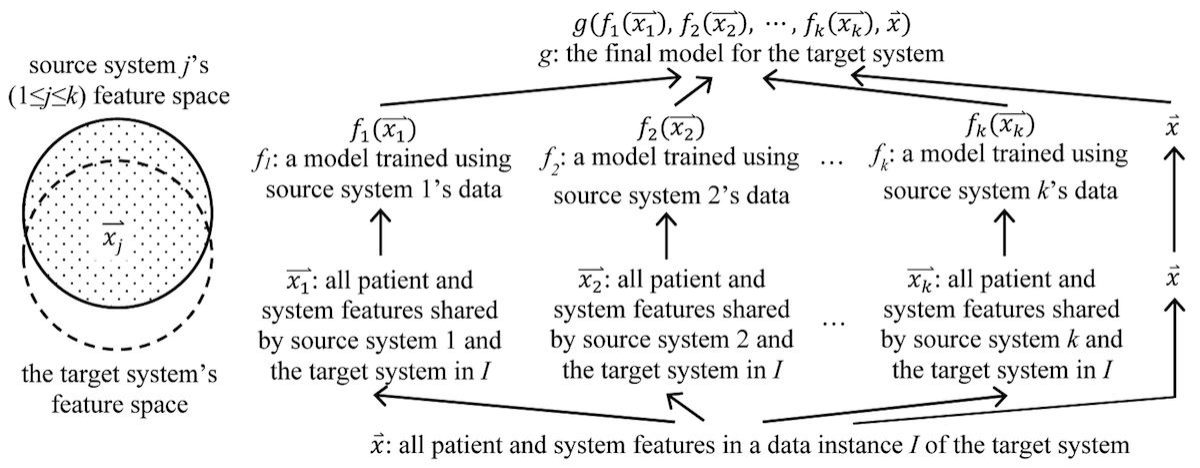
An illustration of our transfer learning approach.

Different health care systems use differing schemas, medical coding systems, and medical terminologies. To enable the application of a model trained using a source system’s data to the target system’s data, we convert the datasets of every source system and the target system into the same common data model (eg, OMOP [85]) format and its linked standardized terminologies [86]. For each available source system, we use the method described in our paper [27] to form a table listing various combinations of attributes. For each combination of attributes, the table includes the model trained using it and the source system’s data. For the combination of attributes collected by both the source and target systems, we find the corresponding model trained for the source system. For every data instance of the target system, we apply the model to the data instance, obtain the prediction result, and append it as a new feature to the data instance. In this way, the expanded data instance includes two types of features: (1) the new features obtained using the models trained for the source systems, with one feature per source system and (2) the patient and system features in the target system. For the target system, we use both types of features and its data to build the final model (Figure 1). As correlation exists among features of the first type constructed for the same prediction target, regularization is likely needed to make the final model stable. Features of the second type can either serve as inputs to the final model directly or be used to build a model whose output serves as an input to the final model. If the target system has limited training data, we perform aggressive feature selection on the second type of features to let the number of remaining features match the amount of training data. This does not impact the first type of features. When a source system has enough data to train a model, the model can include many patient and system features as its inputs. The corresponding feature of the first type is computed using these inputs. In this case, the final model for the target system uses information from many patient and system features, regardless of whether the target system has a large amount of training data.

### Techniques for Creating a New Function to Automatically Explain Prediction Results for Identified High-Risk Patients

To improve prediction accuracy, it is desirable to use machine learning to construct models for predicting individual patient costs and health outcomes [27]. For patients with projected risk above a fixed threshold, such as the 95th percentile, we can use our previously developed method [27,87] to automatically explain machine learning prediction results with no accuracy loss. The explanations can help clinicians make care management enrollment decisions, identify interventions at various levels, create tailored care plans based on objective data, and inspect structured attributes in patient records more efficiently. An example of patient interventions that can be put into tailored care plans is to offer transportation for a patient living far from the primary care physician. An example of interventions at the system level is to launch a new primary care clinic in a region with no such clinic close by.

Each patient has the same set of patient and health care system features and is marked as high or not high risk. Our method mines from historical data class-based association rules related to high risk. Each rule’s left hand side is the conjunction of one or more feature-value pairs. An example rule is as follows: the patient had two or more urgent care visits for asthma last year AND lives 15 miles or more away from the patient’s physician → high risk. Through discussion and consensus, the clinicians in the automatic explanation function’s design team check mined rules and drop those making no or little clinical sense. For every rule kept, the clinicians enumerate zero or more interventions targeting the reason the rule shows. At prediction time, for each patient the predictive model identifies as high risk, we find and display all rules of which the patient fulfills the left hand side conditions. Every rule shows a reason why the patient is projected to be at high risk.

#### Conditional Risk Factors

Our method can find a new type of risk factor termed conditional risk factors, which increase a patient’s risk only when some other variables are also present and can be used to design tailored interventions. This broadens risk factors’ scope, as ordinary risk factors are independent of other variables. Our method can automatically find appropriate cut-off thresholds for numerical variables and inform new interventions based on objective data. For instance, for the aforementioned association rule, our method would automatically find the cut-off thresholds of two in the number of urgent care visits and 15 miles in distance. Then we map all the patients who satisfy the rule’s left hand side conditions and have adverse outcomes in the next year. For the intervention of opening new primary care clinics, this informs the new clinics’ locations by maximizing the number of these patients living less than 15 miles away. A cost-benefit analysis can determine whether adopting this intervention is worthwhile.

#### Use Association Rules to Help Understand the Subtleties in the Data and Improve Model Accuracy

For each association rule related to high risk, the proportion of patients who are at high risk and satisfy the rule’s left hand side is called the rule’s support showing the rule’s coverage. Among all patients fulfilling the rule’s left hand side, the proportion of patients at high risk is called the rule’s confidence showing the rule’s accuracy. Our method discretizes each numerical feature into a categorical one, and mines rules exceeding some predefined minimum support *s*_1_ and minimum confidence *c*_1_ and containing only features that the predictive model uses to make predictions, no more than a preselected number *n*_1_ of feature-value pairs on the left hand side and no feature-value pairs that the automatic explanation function’s designers specify as unrelated to high risk.

Consider all of the association rules related to high risk and satisfying all conditions above except for the last one. If a feature-value pair is specified by the automatic explanation function’s designers as unrelated to high risk but appears in many of these rules, the designers can examine the pair in detail and determine the following [84]:

1. Whether the pair is associated with a surrogate condition related to high risk. This helps us understand the subtleties in the data and how they affect machine learning. Sometimes, we can use the information to design new interventions targeting the surrogate condition. For instance, suppose the pair is that the patient had two outpatient visits for asthma last year and the associated surrogate condition is noncompliance coupled with high vulnerability, for example, because of genetics or working environment. For each rule related to high risk and whose left hand side contains the pair and indicates the surrogate condition (eg, by mentioning that the patient had at least two hospitalizations for asthma last year), we keep the rule, inspect the patients satisfying the rule’s left hand side, and arrange regular phone checks for some of them.
2. Whether the feature is uninformative. Retraining the predictive model after dropping the feature can possibly serve as a new way to improve model accuracy and make the model generalize better to other health care systems beyond the one in which it was developed. Ribeiro et al [88] showed that on nonclinical data, users of an automatic explanation function could use sparse linear model-based explanations to find uninformative features. Retraining the model after dropping these features improved model accuracy. We are unaware of any published work using rule-based explanations to do this, particularly on clinical data. As Ribeiro et al [89] stated, rule-based explanations are preferred over sparse linear model-based ones. The approach by Ribeiro et al [88] works for binary features only. In comparison, our approach can handle all kinds of features.

A health care system often has limited training data impacting model accuracy. To improve model accuracy, we can enlarge the training set by generating synthetic data instances:

1. Using historical data from the target or other source systems, we mine another set *R*_2_ of association rules related to high risk. The clinicians in the automatic explanation function’s design team check the rules in *R*_2_ and keep only those making much clinical sense (eg, tending to generalize across different systems). If desired, we can remove additional rules from *R*_2_ so that the remaining ones are not too similar to each other. For each remaining rule *r* ϵ *R*_2_, we generate multiple synthetic data instances. Each synthetic data instance *I*_s_ satisfies the left hand side of *r* and is labeled high risk. For each feature not on the left hand side of *r*, the feature value of *I*_s_ is chosen randomly. For each numerical feature that our automatic explanation method discretizes into a categorical one, the numerical feature value of *I*_s_ is chosen randomly within the bounds of the category corresponding to the categorical feature value of *I*_s_. Compared with those used for giving explanations, the rules in *R*_2_ are required to exceed some predefined minimum confidence *c*_2_ that is both larger than *c*_1_ and close to 1 (eg, 90%), so that the synthetic data instances are likely to be correctly labeled. To help ensure *R*_2_ contains enough rules, each rule in *R*_2_ needs to exceed a lower predefined minimum support *s*_2_< *s*_1_ and contains no more than a larger, preselected number *n*_2_> *n*_1_ of feature-value pairs on the left hand side.
2. The clinicians specify some rules related to high risk based on medical knowledge. Each rule is used to generate multiple synthetic data instances in a way similar to above. Alternatively, we can use these rules and the predictive model together at prediction time. We use these rules to identify a subset of high-risk patients and apply the predictive model to the other patients not satisfying the left hand side of any of these rules.

Using synthetic data instances to improve model accuracy has been done before, for example, for images [90] or via making interpolations among actual data instances [91]. We are unaware of any published work using association rules for this purpose. In contrast to interpolating all feature values of each synthetic data instance, our association rule-based method retains key feature values to increase the chance that the data instance is correctly labeled.

#### Expand Automatic Explanation’s Coverage of Patients

The mined association rules *R*_1_ used for giving explanations represent frequent patterns linked to high risk. Yet, certain patients are at high risk for uncommon reasons and not covered by any of these rules. To expand automatic explanation’s coverage of patients, we improve our prior method [27,87] by generating synthetic data instances, adopting the predictive model to label them, and using them to mine additional rules to cover more patients [88]. The improved method generalizes to many clinical applications.

More specifically, during association rule mining, some rules are found and then removed because they fall below the predefined minimum support *s*_1_ or minimum confidence *c*_1_. Instead of removing them, we keep as backup all such rules *R*_3_ that exceed both the minimum confidence *c*_1_ and another predefined minimum support *s*_3_< *s*_1_ and sort them in descending order of support. We can use techniques similar to those used in our prior method [27,87] to prune redundant rules in *R*_3_. At prediction time, for each patient the predictive model identifies as high risk and not covered by any rule in the set *R*_1_, we check the rules in the backup set *R*_3_ sequentially. For each rule *r* ϵ *R*_3_, we generate one or more synthetic data instances in a way similar to above to make the total number of data instances satisfying *r* ’s left hand side reach the minimum support *s*_1_. We use the predictive model to make predictions on and label the synthetic data instances. Using both the synthetic data instances and data instances in the training set satisfying *r* ’s left hand side, we check whether *r* exceeds the minimum confidence *c*_1_. If so, we stop the rule checking process and display *r* as the automatically generated explanation for the patient. Otherwise, we continue to check the next rule in *R*_3_. The predictive model may make incorrect predictions on and mislabel some synthetic data instances, causing the finally chosen rule to not reflect the true reason why the patient is at high risk. By sorting the rules in *R*_3_ in descending order of support, we minimize the number of synthetic data instances to be generated for the finally chosen rule and reduce this likelihood.

Unlike the rules in the set *R*_1_, the rules in the backup set *R*_3_ are not prechecked by the automatic explanation function’s design team. Some rules in *R*_3_ may make no or little clinical sense. At prediction time, users of the automatic explanation function can provide feedback on the displayed rules chosen from *R*_3_. This helps the automatic explanation function’s design team identify unreasonable rules and remove them from *R*_3_ so that they will not be displayed in the future. For example, if the number of times that a rule in *R*_3_ has been displayed to users exceeds a given threshold and the proportion of times that users report the rule as unreasonable is over a fixed limit, the rule can become a candidate for removal from *R*_3_.

### Technique for Making Causal Inference for Periodically Reidentifying High-Risk Patients

To provide causal inference capability, we need to estimate the impact of care management on a patient’s cost or health outcome. We use this estimate to adjust the cost or health outcome threshold for deciding whether a patient on care management should be moved off care management. Propensity score matching is one technique for doing this on observational data [80,81,92]. Using the same features adopted for predicting individual patient cost or health outcome, we build a model to predict whether a patient will be enrolled in care management. The propensity score is the predicted probability of enrollment. We match each patient on care management to a patient not on care management on propensity score. The impact of care management is estimated as the average cost or health outcome difference between the group of patients on care management and the matched group of patients not on care management. We can apply the propensity score matching technique to the entire group of patients. Alternatively, if we expect care management to have greatly varying impact across different subgroups of patients, we can apply the propensity score matching technique to each subgroup of patients separately.

## Conclusions

Care management is broadly used for improving asthma outcomes and cutting costs, but current high-risk patient identification methods have major limitations degrading its effectiveness. This paper pinpoints these limitations and outlines multiple machine learning techniques to address them, offering a roadmap for future research. Besides being used for asthma, care management is also broadly adopted in managing patients with diabetes, heart diseases, or chronic obstructive pulmonary disease [5], where similar limitations in patient identification exist and techniques similar to those outlined in this paper can be used to optimize care management. The principles of many of our proposed techniques generalize to other predictive modeling tasks beyond those for care management.

## Abbreviations

EMR: electronic medical record

## References

[1] CDC.

[2] Akinbami LJ, Moorman JE, Liu X (2011). Asthma prevalence, health care use, and mortality: United States, 2005-2009. Natl Health Stat Report.

[3] Akinbami LJ, Moorman JE, Bailey C, Zahran HS, King M, Johnson CA, Liu X (2012). Trends in asthma prevalence, health care use, and mortality in the United States, 2001-2010. NCHS Data Brief.

[4] CDC.

[5] Duncan I (2011). Healthcare Risk Adjustment and Predictive Modeling.

[6] Schatz M, Nakahiro R, Jones CH, Roth RM, Joshua A, Petitti D (2004). Asthma population management: development and validation of a practical 3-level risk stratification scheme. Am J Manag Care.

[7] Axelrod RC, Vogel D (2003). Predictive modeling in health plans. Disease Management & Health Outcomes.

[8] Vogeli C, Shields AE, Lee TA, Gibson TB, Marder WD, Weiss KB, Blumenthal D (2007). Multiple chronic conditions: prevalence, health consequences, and implications for quality, care management, and costs. J Gen Intern Med.

[9] Caloyeras JP, Liu H, Exum E, Broderick M, Mattke S (2014). Managing manifest diseases, but not health risks, saved PepsiCo money over seven years. Health Aff (Millwood).

[10] Nelson L CBO.

[11] Ccmcertification.

[12] Kelly CS, Morrow AL, Shults J, Nakas N, Strope GL, Adelman RD (2000). Outcomes evaluation of a comprehensive intervention program for asthmatic children enrolled in medicaid. Pediatrics.

[13] Forno E, Celedón JC (2012). Predicting asthma exacerbations in children. Curr Opin Pulm Med.

[14] Levine S, Adams J, Attaway K, Dorr D, Leung M, Popescu P, Rich J CHCF.

[15] Rubin RJ, Dietrich KA, Hawk AD (1998). Clinical and economic impact of implementing a comprehensive diabetes management program in managed care. J Clin Endocrinol Metab.

[16] Greineder DK, Loane KC, Parks P (1999). A randomized controlled trial of a pediatric asthma outreach program. J Allergy Clin Immunol.

[17] Axelrod R, Zimbro K, Chetney R, Sabol J, Ainsworth V (2001). A disease management program utilizing life coaches for children with asthma. J Clin Outcomes Manag.

[18] Dorr DA, Wilcox AB, Brunker CP, Burdon RE, Donnelly SM (2008). The effect of technology-supported, multidisease care management on the mortality and hospitalization of seniors. J Am Geriatr Soc.

[19] Beaulieu N, Cutler D, Ho K, Isham G, Lindquist T, Nelson A, O'Connor P (2006). The business case for diabetes disease management for managed care organizations. Forum Health Econ Policy.

[20] Curry N, Billings J, Darin B, Dixon J, Williams M, Wennberg D Kingsfund.

[21] Moturu S, Johnson W, Liu H (2007). Predicting future high-cost patients: a real-world risk modeling application. Proceedings of the IEEE International Conference on Bioinformatics and Biomedicine.

[22] Fleishman JA, Cohen JW, Manning WG, Kosinski M (2006). Using the SF-12 health status measure to improve predictions of medical expenditures. Med Care.

[23] Bertsimas D, Bjarnadóttir MV, Kane MA, Kryder JC, Pandey R, Vempala S, Wang G (2008). Algorithmic prediction of health-care costs. Operations Research.

[24] Anderson RT, Balkrishnan R, Camacho F (2004). Risk classification of Medicare HMO enrollee cost levels using a decision-tree approach. Am J Manag Care.

[25] Meenan RT, Goodman MJ, Fishman PA, Hornbrook MC, O'Keeffe-Rosetti MC, Bachman DJ (2003). Using risk-adjustment models to identify high-cost risks. Med Care.

[26] Weir S, Aweh G, Clark RE (2008). Case selection for a Medicaid chronic care management program. Health Care Financ Rev.

[27] Luo G, Stone BL, Sakaguchi F, Sheng X, Murtaugh MA (2015). Using computational approaches to improve risk-stratified patient management: rationale and methods. JMIR Res Protoc.

[28] Hibbard JH, Greene J, Sacks RM, Overton V, Parrotta C (2017). Improving population health management strategies: identifying patients who are more likely to be users of avoidable costly care and those more likely to develop a new chronic disease. Health Serv Res.

[29] Greene J, Hibbard JH, Sacks R, Overton V, Parrotta CD (2015). When patient activation levels change, health outcomes and costs change, too. Health Aff (Millwood).

[30] Fogg B (2009). A behavior model for persuasive design. Proceedings of the International Conference on Persuasive Technology.

[31] Naylor MD, Bowles KH, McCauley KM, Maccoy MC, Maislin G, Pauly MV, Krakauer R (2013). High-value transitional care: translation of research into practice. J Eval Clin Pract.

[32] Diehr P, Yanez D, Ash A, Hornbrook M, Lin DY (1999). Methods for analyzing health care utilization and costs. Annu Rev Public Health.

[33] Ash A, McCall N RTI.

[34] Iezzoni L (2013). Risk Adjustment for Measuring Health Care Outcomes. 4th edition.

[35] Neuvirth H, Ozery-Flato M, Hu J, Laserson J, Kohn M, Ebadollahi S, Rosen-Zvi M (2011). Toward personalized care management of patients at risk: the diabetes case study. Proceedings of the ACM SIGKDD International Conference on Knowledge Discovery and Data Mining.

[36] Schatz M, Cook EF, Joshua A, Petitti D (2003). Risk factors for asthma hospitalizations in a managed care organization: development of a clinical prediction rule. Am J Manag Care.

[37] Lieu TA, Quesenberry CP, Sorel ME, Mendoza GR, Leong AB (1998). Computer-based models to identify high-risk children with asthma. Am J Respir Crit Care Med.

[38] Lieu TA, Capra AM, Quesenberry CP, Mendoza GR, Mazar M (1999). Computer-based models to identify high-risk adults with asthma: is the glass half empty or half full?. J Asthma.

[39] Forno E, Fuhlbrigge A, Soto-Quirós ME, Avila L, Raby BA, Brehm J, Sylvia JM, Weiss ST, Celedón JC (2010). Risk factors and predictive clinical scores for asthma exacerbations in childhood. Chest.

[40] Miller MK, Lee JH, Blanc PD, Pasta DJ, Gujrathi S, Barron H, Wenzel SE, Weiss ST (2006). TENOR risk score predicts healthcare in adults with severe or difficult-to-treat asthma. Eur Respir J.

[41] Razavian N, Blecker S, Schmidt AM, Smith-McLallen A, Nigam S, Sontag D (2015). Population-level prediction of type 2 diabetes from claims data and analysis of risk factors. Big Data.

[42] Schatz M (2012). Predictors of asthma control: what can we modify?. Curr Opin Allergy Clin Immunol.

[43] Stanford RH, Shah MB, D'Souza AO, Schatz M (2013). Predicting asthma outcomes in commercially insured and Medicaid populations. Am J Manag Care.

[44] Hyland ME, Whalley B, Halpin DM, Greaves CJ, Seamark C, Blake S, Pinnuck M, Ward D, Hawkins A, Seamark D (2012). Frequency of non-asthma GP visits predicts asthma exacerbations: an observational study in general practice. Prim Care Respir J.

[45] Crawford AG, Fuhr JP, Clarke J, Hubbs B (2005). Comparative effectiveness of total population versus disease-specific neural network models in predicting medical costs. Dis Manag.

[46] Coyle YM (2003). Predictors of acute asthma relapse: strategies for its prevention. J Asthma.

[47] Evans RS (2016). Electronic health records: then, now, and in the future. Yearb Med Inform.

[48] Sun J, Hu J, Luo D, Markatou M, Wang F, Edabollahi S, Steinhubl SE, Daar Z, Stewart WF (2012). Combining knowledge and data driven insights for identifying risk factors using electronic health records. AMIA Annu Symp Proc.

[49] Phillips CD, Chen M, Sherman M (2008). To what degree does provider performance affect a quality indicator? the case of nursing homes and ADL change. Gerontologist.

[50] Fung V, Schmittdiel JA, Fireman B, Meer A, Thomas S, Smider N, Hsu J, Selby JV (2010). Meaningful variation in performance: a systematic literature review. Med Care.

[51] Lipitz-Snyderman A, Sima CS, Atoria CL, Elkin EB, Anderson C, Blinder V, Tsai CJ, Panageas KS, Bach PB (2016). Physician-driven variation in nonrecommended services among older adults diagnosed with cancer. JAMA Intern Med.

[52] Obermeyer Z, Powers BW, Makar M, Keating NL, Cutler DM (2015). Physician characteristics strongly predict patient enrollment in hospice. Health Aff (Millwood).

[53] Borsi JP (2016). Hypothesis-free search for connections between birth month and disease prevalence in large, geographically varied cohorts. AMIA Annu Symp Proc.

[54] Wiens J, Guttag J, Horvitz E (2014). A study in transfer learning: leveraging data from multiple hospitals to enhance hospital-specific predictions. J Am Med Inform Assoc.

[55] Singleton KW, Hsu W, Bui AAT (2012). Comparing predictive models of glioblastoma multiforme built using multi-institutional and local data sources. AMIA Annu Symp Proc.

[56] Bleeker SE, Moll HA, Steyerberg EW, Donders AR, Derksen-Lubsen G, Grobbee DE, Moons KG (2003). External validation is necessary in prediction research: a clinical example. J Clin Epidemiol.

[57] Siontis GC, Tzoulaki I, Castaldi PJ, Ioannidis JP (2015). External validation of new risk prediction models is infrequent and reveals worse prognostic discrimination. J Clin Epidemiol.

[58] Gong J, Sundt T, Rawn J, Guttag J (2015). Instance weighting for patient-specific risk stratification models. Proceedings of the ACM SIGKDD International Conference on Knowledge Discovery and Data Mining.

[59] Lee G, Rubinfeld I, Syed Z (2012). Adapting surgical models to individual hospitals using transfer learning. Proceedings of IEEE International Conference on Data Mining Workshops.

[60] Pan SJ, Yang Q (2010). A survey on transfer learning. IEEE Trans Knowl Data Eng.

[61] Weiss K, Khoshgoftaar TM, Wang D (2016). A survey of transfer learning. J Big Data.

[62] Jayanthi A Beckershospitalreview.

[63] Hripcsak G, Duke JD, Shah NH, Reich CG, Huser V, Schuemie MJ, Suchard MA, Park RW, Wong IC, Rijnbeek PR, van DL, Pratt N, Norén GN, Li Y, Stang PE, Madigan D, Ryan PB (2015). Observational health data sciences and informatics (OHDSI): opportunities for observational researchers. Stud Health Technol Inform.

[64] Fleurence RL, Curtis LH, Califf RM, Platt R, Selby JV, Brown JS (2014). Launching PCORnet, a national patient-centered clinical research network. J Am Med Inform Assoc.

[65] Steyerberg E (2009). Clinical Prediction Models: A Practical Approach to Development, Validation, and Updating.

[66] Zhou Z (2012). Ensemble Methods: Foundations and Algorithms.

[67] Gao J, Fan W, Jiang J, Han J (2008). Knowledge transfer via multiple model local structure mapping. Proceedings of the ACM SIGKDD International Conference on Knowledge Discovery and Data Mining.

[68] Halamka JD (2014). Early experiences with big data at an academic medical center. Health Aff (Millwood).

[69] Miller GA (1956). The magical number seven plus or minus two: some limits on our capacity for processing information. Psychol Rev.

[70] Luo G (2014). A roadmap for designing a personalized search tool for individual healthcare providers. J Med Syst.

[71] James BC, Savitz LA (2011). How Intermountain trimmed health care costs through robust quality improvement efforts. Health Aff (Millwood).

[72] Dartmouthatlas.

[73] Pacific Business Group on Health PBGH.

[74] Gifford E, Foster EM (2008). Provider-level effects on psychiatric inpatient length of stay for youth with mental health and substance abuse disorders. Med Care.

[75] Kramer TL, Daniels AS, Zieman GL, Williams C, Dewan NA (2000). Psychiatric practice variations in the diagnosis and treatment of major depression. Psychiatr Serv.

[76] Iwashyna TJ, Chang VW, Zhang JX, Christakis NA (2002). The lack of effect of market structure on hospice use. Health Serv Res.

[77] Barnett ML, Olenski AR, Jena AB (2017). Opioid-prescribing patterns of emergency physicians and risk of long-term use. N Engl J Med.

[78] Keating NL, Herrinton LJ, Zaslavsky AM, Liu L, Ayanian JZ (2006). Variations in hospice use among cancer patients. J Natl Cancer Inst.

[79] Orueta JF, García-Alvarez A, Grandes G, Nuño-Solinís R (2015). The origin of variation in primary care process and outcome indicators: patients, professionals, centers, and health districts. Medicine (Baltimore).

[80] Morgan S, Winship C (2014). Counterfactuals and Causal Inference: Methods and Principles for Social Research. 2nd edition.

[81] Pearl J (2009). Causality: Models, Reasoning, and Inference. 2nd edition.

[82] Luo D, Wang F, Sun J, Markatou M, Hu J, Ebadollahi S (2012). SOR: Scalable orthogonal regression for low-redundancy feature selection and its healthcare applications. Proceedings of the SIAM International Conference on Data Mining.

[83] Hall ES, Thornton SN (2008). Generating nurse profiles from computerized labor and delivery documentation. AMIA Annu Symp Proc.

[84] Schuit E, Groenwold RH, Harrell FE, de Kort WL, Kwee A, Mol BW, Riley RD, Moons KG (2013). Unexpected predictor-outcome associations in clinical prediction research: causes and solutions. CMAJ.

[85] OMOP.

[86] OMOP.

[87] Luo G (2016). Automatically explaining machine learning prediction results: a demonstration on type 2 diabetes risk prediction. Health Inf Sci Syst.

[88] Ribeiro M, Singh S, Guestrin C (2016). “Why Should I Trust You?”: explaining the predictions of any classifier. Proceedings of the ACM SIGKDD International Conference on Knowledge Discovery and Data Mining.

[89] Ribeiro M, Singh S, Guestrin C (2016). Nothing else matters: model-agnostic explanations by identifying prediction invariance. Proceedings of NIPS Workshop on Interpretable Machine Learning in Complex Systems.

[90] Nonnemaker J, Baird H (2009). Using synthetic data safely in classification. Proceedings of Document Recognition and Retrieval XVI of the IS&T-SPIE Electronic Imaging Symposium.

[91] Chawla N, Bowyer K, Hall L, Kegelmeyer W (2002). SMOTE: synthetic minority over-sampling technique. J Artif Intell Res.

[92] Duncan I (2014). Managing and Evaluating Healthcare Intervention Programs. 2nd edition.

